# LCFC-Laptop: A Benchmark Dataset for Detecting Surface Defects in Consumer Electronics

**DOI:** 10.3390/s25154535

**Published:** 2025-07-22

**Authors:** Hua-Feng Dai, Jyun-Rong Wang, Quan Zhong, Dong Qin, Hao Liu, Fei Guo

**Affiliations:** 1 Department of Mechanical Engineering, Tsinghua University, Beijing 100084, China; huafeng.dai@lcfuturecenter.com; 2 LCFC (Hefei) Electronics Technology Co., Ltd., Hefei 230088, China; bengdex.wang@lcfuturecenter.com (J.-R.W.); quan.zhong@lcfuturecenter.com (Q.Z.); dong.qin@lcfuturecenter.com (D.Q.); hao.liu@lcfuturecenter.com (H.L.); 3 Hefei LCFC Information Technology Co., Ltd., Hefei 230088, China; 4 School of Computer Science and Technology, University of Science and Technology of China, Hefei 230000, China

**Keywords:** surface defect detection in consumer electronics, segmentation, object detection, dataset of surface defects in consumer electronics

## Abstract

As a high-market-value sector, the consumer electronics industry is particularly vulnerable to reputational damage from surface defects in shipped products. However, the high level of automation and the short product life cycles in this industry make defect sample collection both difficult and inefficient. This challenge has led to a severe shortage of publicly available, comprehensive datasets dedicated to surface defect detection, limiting the development of targeted methodologies in the academic community. Most existing datasets focus on general-purpose object categories, such as those in the COCO and PASCAL VOC datasets, or on industrial surfaces, such as those in the MvTec AD and ZJU-Leaper datasets. However, these datasets differ significantly in structure, defect types, and imaging conditions from those specific to consumer electronics. As a result, models trained on them often perform poorly when applied to surface defect detection tasks in this domain. To address this issue, the present study introduces a specialized optical sampling system with six distinct lighting configurations, each designed to highlight different surface defect types. These lighting conditions were calibrated by experienced optical engineers to maximize defect visibility and detectability. Using this system, 14,478 high-resolution defect images were collected from actual production environments. These images cover more than six defect types, such as scratches, plain particles, edge particles, dirt, collisions, and unknown defects. After data acquisition, senior quality control inspectors and manufacturing engineers established standardized annotation criteria based on real-world industrial acceptance standards. Annotations were then applied using bounding boxes for object detection and pixelwise masks for semantic segmentation. In addition to the dataset construction scheme, commonly used semantic segmentation methods were benchmarked using the provided mask annotations. The resulting dataset has been made publicly available to support the research community in developing, testing, and refining advanced surface defect detection algorithms under realistic conditions. To the best of our knowledge, this is the first comprehensive, multiclass, multi-defect dataset for surface defect detection in the consumer electronics domain that provides pixel-level ground-truth annotations and is explicitly designed for real-world applications.

## 1. Introduction

The global consumer electronics market reached USD 6.9 billion in 2023 and is projected to grow to USD 9.8 billion by 2031, with a compound annual growth rate (CAGR) of 4.1% during the forecasting period from 2024 to 2031 [1]. This steady growth highlights the increasing demand for high-quality products and the importance of maintaining strong brand reputations. Surface defects in shipped products can significantly damage a manufacturer’s reputation and erode customer trust. Consequently, effective surface defect detection is critical to quality control. However, many general-purpose detection and segmentation methods often perform poorly when addressing the specific challenges of surface defect detection in consumer electronics.

Deep learning-based methods have been successfully applied across various domains in recent years [2,3,4,5,6,7,8], demonstrating excellent predictive performance when sufficient training data are available [9,10,11]. Based on the specific application requirements, these models are typically categorized into distinct computer vision tasks, including object detection [12], semantic segmentation, small-object detection [13], and real-time detection [14]. Moreover, researchers have proposed various improvements to baseline models by exploring novel regularization techniques, customized loss functions, neural network architectural innovations [15], and alternative feature extraction strategies [16,17]. In the context of surface defect detection in consumer electronics, bi-objective optimization is especially important, as both prediction accuracy and inference speed must be considered. Methods that achieve high accuracy but incur long inference times are unsuitable due to the stringent time constraints imposed by industrial production cycles.

Publicly accessible datasets include general-purpose object detection datasets such as the COCO dataset [18] and the PASCAL VOC dataset [19], as well as commonly used industrial datasets such as MvTec AD [20] and ZJU-Leaper [21]. Consumer electronics defects often involve fine-grained surface variations, subtle texture inconsistencies, and category ambiguity under varying illumination—characteristics that differ significantly from the typically larger and more structured defects found in sectors such as automotive or textile manufacturing. In addition, surface defect datasets in consumer electronics present specific challenges, including ill-defined defect types, low contrast between defects and backgrounds, and considerable variation in defect scales. These characteristics make it challenging for general-purpose detection and segmentation algorithms to produce satisfactory results on such datasets.

Moreover, the high level of automation and short product life cycles in consumer electronics limit the collection of sufficient defect samples, resulting in a lack of publicly available, comprehensive datasets specifically designed for surface defect detection. Beyond dataset-specific challenges, surface defect detection in consumer electronics also requires models that are lightweight, capable of processing high-resolution inputs, and easy to deploy. These requirements are essential for real-world implementation, where models must meet strict production cycle-time constraints.

Numerous studies have proposed novel methods for industrial defect detection. For example, as discussed in [22,23,24], various deep learning-based approaches have achieved promising results across different industrial domains and may serve as valuable references for readers exploring this field. However, as previously mentioned, surface defect detection in consumer electronics differs significantly from conventional industrial applications. Consequently, existing methods often require adaptation or modification to perform effectively on consumer electronics surface defect datasets.

To address this gap, we introduce a consumer electronics surface defect detection dataset that includes both object detection and segmentation annotations. These annotations follow strict production line standards and were repeatedly verified by professional quality inspectors to ensure accuracy. To enable pixel-level evaluation, semantic segmentation was selected as the primary assessment task. Semantic segmentation methods can be broadly categorized into transformer-based and convolutional neural network (CNN)-based approaches. Transformer-based methods, such as those proposed in [25,26,27,28,29], generally prioritize accuracy. CNN-based methods, which offer advantages in inference speed, have also been extensively studied in recent years [29,30,31,32,33,34]. Moreover, hybrid models that integrate both convolutional and transformer architectures have emerged, yielding promising results across various tasks [35,36,37,38,39]. However, in the context of surface defect detection for consumer electronics, the stringent requirements for real-time inference impose significant constraints. Therefore, given the unique characteristics of this task, the dataset was evaluated using four commonly employed convolution-based segmentation networks [40,41,42,43], providing a valuable reference for both academic and industrial communities. The goal is to support the development of customized algorithms designed to address the specific challenges of surface defect detection in consumer electronics, thereby promoting practical and efficient solutions in real-world manufacturing environments.

Traditional industrial surface defect detection methods can be broadly categorized into two groups. The first consists of traditional machine vision methods, which rely on hand-crafted features, including texture features (e.g., Gabor filters, Gray-Level Co-occurrence Matrix (GLCM), and Local Binary Patterns (LBPs)), color features, (e.g., color contrast and histogram-based descriptors), and shape features (e.g., edge detection, contour extraction, and geometric descriptors). The second group consists of deep learning-based methods, further divided by the availability of labeled data into supervised, unsupervised, and weakly supervised approaches. Despite their differences, both categories face common challenges, including real-time inference requirements, detection of small defects, class imbalance, and, most critically, the limited availability of annotated samples, which makes small-sample learning a critical issue in practical applications.

## 2. Materials and Methods

Due to the substantial differences between consumer electronics surface defect datasets and general-purpose publicly available benchmark datasets, the latter cannot effectively evaluate algorithm performance on real-world data. For example, in consumer electronics defect detection, target objects often lack clearly defined appearances, exhibit varied size distributions, and present challenges distinct from those in standard datasets. As a result, open-source benchmarks fail to provide reliable assessments of real-world algorithm performance. To ensure fair comparisons among different algorithms, this section provides a brief introduction to the benchmark datasets used in the current study. Additionally, a consumer electronics surface defect dataset containing rare defect cases was constructed using real industrial data. This dataset was meticulously labeled over an extended period using a multi-light-source, multi-camera system, making it a valuable resource for evaluating algorithmic performance in real-world industrial applications.

### 2.1. LCFC-Laptop Dataset

LCFC (Hefei) Electronics Technology Co., Ltd. (LCFC) is a wholly owned subsidiary of Lenovo. Of every eight laptops sold globally, one is manufactured by LCFC. The company has sold over 200 million laptops, with Lenovo laptops purchased in more than 126 countries. Through collaboration with LCFC, we established a comprehensive laptop dataset to evaluate the performance of the proposed algorithm.

The LCFC-Laptop dataset was constructed using samples captured by four high-resolution industrial cameras, each with a resolution of 5000 × 5000 pixels. Each camera was equipped with six lighting sources, including white and blue light. We hypothesized that certain defects correlate with specific wavelengths and that broad-spectrum illumination could capture a wider range of defect types. Therefore, light sources of different wavelengths were employed, with all six lighting configurations carefully calibrated by experienced optical engineers. The dataset was annotated according to standards defined by LCFC’s senior engineers and quality inspectors.

The dataset primarily includes the following four categories:

1. Scratches: Visible scratches or scuff marks on laptop surfaces.
2. Collisions: Damage caused by impacts or other physical factors, such as dents or cracks.
3. Plain particles: Normal surfaces with minor dust or debris, considered minor defects that affect surface appearance but not functionality.
4. Dirt: Stains, fingerprints, or other forms of contamination on laptop surfaces that affect cleanliness.

**Figure 1:**
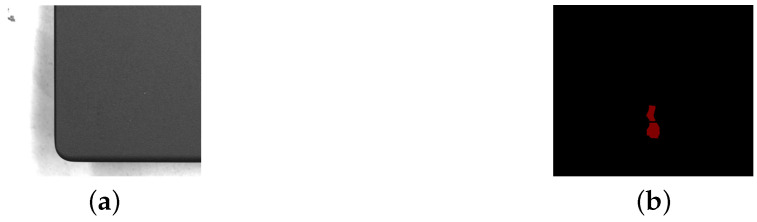
An original image and the corresponding labeled image of dirt. (**a**) Original image. (**b**) Labeled image. The scale is 1 mm = 25 pixels.

**Figure 2:**
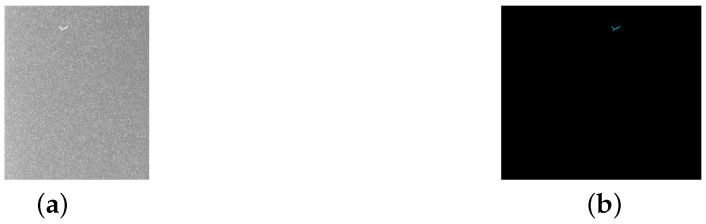
An original image and the corresponding labeled image of a scratch. (**a**) Original image. (**b**) Labeled image. The scale is 1 mm = 25 pixels.

**Figure 3:**
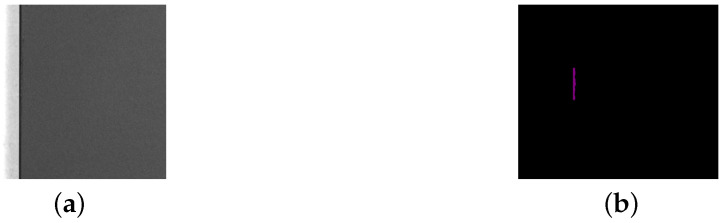
An original image and the corresponding labeled image of a collision. (**a**) Original image. (**b**) Labeled image. The scale is 1 mm = 25 pixels.

**Figure 4:**
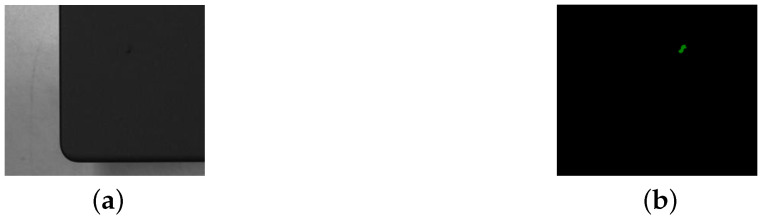
An original image and the corresponding labeled image of a plain particle. (**a**) Original image. (**b**) Labeled image. The scale is 1 mm = 25 pixels.

Examples of each defect type are shown in Figure 1, Figure 2, Figure 3 and Figure 4.

This section also provides a tabular comparison between the LCFC-Laptop dataset and the MVTec AD dataset, as shown in Table 1. A fair comparison is conducted across several key aspects, including defect sample count, number of lighting sources, application domain, annotation type, evaluation protocol, and image resolution.

The LCFC-Laptop dataset contributes to the research community in the following ways:

1. Improving defect detection accuracy: The dataset includes a diverse range of defect samples, enabling the training of models capable of accurately identifying surface defects on laptops, thereby reducing false positives and false negatives.
2. Enhancing automated production line inspection: The dataset serves as essential training data for industrial automation systems, increasing detection speed and accuracy while reducing the costs and errors associated with manual inspection.
3. Advancing AI applications in industry: The dataset supports the development of AI technologies in industrial contexts, particularly for product quality control and visual inspection, thereby improving overall production efficiency and quality.
4. Accelerating smart manufacturing: By leveraging this dataset, manufacturers can develop more efficient automated quality control systems, further driving the digital transformation of the manufacturing industry.

#### 2.1.1. Statistics for the Object Detection Task

The object detection annotations for the constructed dataset are summarized in Table 2. The dataset includes a total of 14,478 defects.

The ‘unknown’ category refers to defects that are visually distinguishable from normal areas but cannot be clearly assigned to a specific defect type. The edge particle category includes particles located along surface edges. Although also considered particle defects, their appearance sometimes differs from that of plain particles.

**Figure 5:**
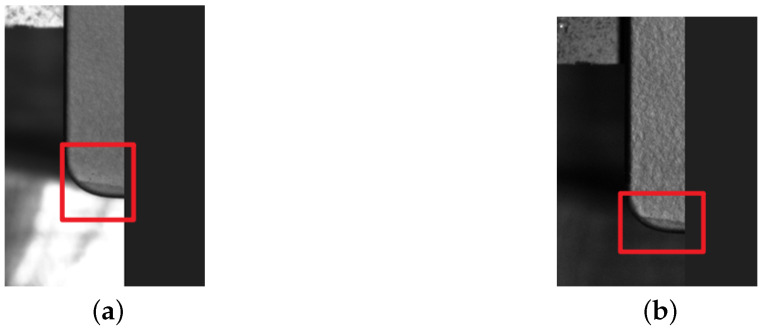
The unknown defects shown do not belong to any existing defect categories. They are typically caused by physical wear, mechanical abrasion, impact, or compression that damages or distorts the surface material. These defects differ clearly from the predefined categories and cannot be assigned to any known defect type. Moreover, the shapes and appearances of these unknown defects vary significantly, and their quantities are uncertain, making it impractical to establish a unified category. (**a**) Unknown defect sample 1. (**b**) Unknown defect sample 2. The red boxes indicate the specific defect regions.

As shown in Figure 5, defects outside the scope of existing classification standards are frequently observed due to the unpredictable nature of defect occurrences. The defects illustrated in the figure are typically caused by physical wear, mechanical abrasion, impact, or compression that damages or deforms the surface material. These defects differ clearly from the predefined categories and cannot be assigned to any known defect type.

#### 2.1.2. Statistics for the Segmentation Task

For the segmentation annotations in this dataset, samples were collected from products made of the same material at different times. Since segmentation involves pixel-level labeling with relatively high granularity, defect regions that are not closely connected are annotated as separate defects. As a result, the number of defects identified in the segmentation task is generally greater than in the object detection task. The segmentation annotations for the constructed dataset are summarized in Table 3. The dataset includes a total of 22,512 defects, categorized as follows: 2572 dirt defects, 4788 plain-particle defects, 5265 collision defects, and 9868 scratch defects.

#### 2.1.3. Challenges Related to the LCFC-Laptop Dataset

In object detection and segmentation tasks, defects smaller than a certain area are referred to as small objects. Specifically, in object detection scenarios, defects occupying areas smaller than 32 × 32 pixels are classified as small objects. In the constructed dataset, particularly in categories such as edge particles, not only are the defect areas small, but the number of samples is also significantly lower than in other categories. As a result, none of the four segmentation methods evaluated in this study were able to effectively predict these defects.

The spatial distribution of the defects shows no significant positional bias, as they appear relatively evenly across different regions of the image. In terms of size, the defects exhibit typical small-object characteristics. More than 60% of the defect bounding boxes have widths under 24 pixels, and more than 30% have heights under 12 pixels. Among these, collision and plain-particle defects are particularly small, averaging approximately half the size of dirt-type defects. However, these smaller defects occur more frequently than dirt-type defects, which further impacts the overall performance of neural networks on the dataset.

Due to the side variation in size distributions within a single class, surface defect detection faces the following challenges:

1. Model bias toward large-area targets: Loss functions such as cross-entropy and Dice loss are typically influenced by the number of pixels. As a result, large-area classes contribute more to the total loss, making the model more likely to learn these categories effectively. This reduces prediction accuracy for small-area targets, which may even be completely overlooked—a phenomenon known as being “overwhelmed” or “dominated by the head class”.
2. Feature loss in small targets: The features of small defects, such as edges or textures, are often blurred or lost through multiple convolutional and downsampling layers in a feature pyramid, making it difficult for the model to recognize tiny abnormal regions. These issues are especially prominent in applications such as consumer electronics, where defects such as microcracks or dust particles are very small.
3. Annotation sensitivity: For small-area targets, even slight annotation misalignments can cause a significant drop in the mean intersection over union (mIoU). This is particularly critical in pixel-level tasks, where high precision is required to segment small objects accurately.

### 2.2. Optical Sampling System

An optical sampling system comprises several key components, including the choice of lighting method, light-source wavelength, and incident angle.

Since the optical properties of different materials vary, adjustments must be made based on the material and defect characteristics of the inspected items. High-angle bright-field illumination enhances surface details and is suitable for detecting defects such as particles and stains. In contrast, low-angle dark-field illumination emphasizes edges and contours, making it more effective for detecting defects such as scratches and collision marks. Therefore, this system incorporates both bright-field and dark-field lighting methods.

Once the lighting method is selected, the appropriate light-source color must be chosen based on the material. As manufacturing materials are relatively consistent, targeted validation is feasible. Commonly used light sources include the following:

1. White light: Suitable for detecting color abnormalities but has limited resistance to ambient light interference.
2. Red light: Ideal for detecting internal defects.
3. Blue light: Effective for identifying microcracks and surface burrs.

**Figure 6:**
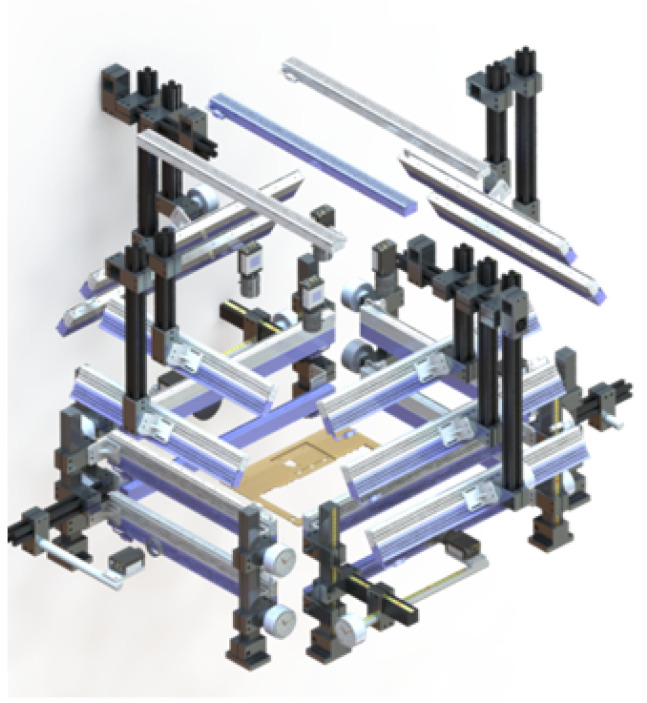
Spatial configuration of the six-directional lighting system, including white, red, and blue light sources positioned at varying incident angles and heights. This multi-illumination setup is designed to simulate diverse lighting conditions and maximize coverage of different surface defect types.

The incident angle of the light source also plays a crucial role in the final image quality. The selected angles include 0°, 30°, 45°, 60°, and 90°, as illustrated in Figure 6. Some defects may not be visible under direct frontal illumination; however, changing the incident angle can make them clearly identifiable. In this study, a senior optical engineer with extensive industry experience performed hyperparameter optimization of the optical sampling system, focusing on three aspects: lighting method, light-source wavelength, and incident angle.

The illumination system proposed in this study can be adapted to different materials within the current design by adjusting parameters with the assistance of experienced optical engineers. For products made of the same material but from different models, practical experience indicates that the same parameter settings can typically be applied without modification.

The six lighting combinations comprise 35 specific white and blue light sources with varying incident angles and model numbers, each corresponding to a designated position in the PCL system. The detailed configuration is shown in Table 4. Additionally, the wavelengths and brightness levels of the lighting sources are provided in Table 5.

### 2.3. Annotation of the LCFC-Laptop Dataset

The first challenge in dataset annotation is establishing a consistent standard. When defect images are ambiguous, different observers may interpret them differently. One engineer might identify a defect, whereas another may classify the image as a normal sample. Additionally, certain defects are difficult to identify without relevant experience.

To ensure high annotation accuracy, multiple senior production line engineers were assigned to conduct the annotation process. Each defect sample was annotated and repeatedly reviewed by experienced engineers, and only those with unanimous agreement were included in the dataset. Furthermore, samples visible to the naked eye but not accurately captured by the camera were excluded to improve overall dataset quality.

To further enhance reliability, an independent group of engineers performed repeated error correction and verification to identify and rectify mislabeling or omissions. This iterative process ensured a high-quality dataset with minimal annotation inconsistencies.

Although every effort was made to annotate all defects, their occurrence is inherently unpredictable. As a result, some clearly visible defects fall outside the predefined categories such as dirt, scratches, or particles. As shown in Figure 5, these defects are visually evident but remain beyond the scope of the currently defined defect types.

### 2.4. Data Evaluation

In this section, we evaluate the constructed dataset using a segmentation-based approach. Since segmentation involves pixelwise classification, it enables finer distinctions in defect detection. However, it also requires significantly more computational resources than object detection methods. Therefore, we used segmentation tasks to assess the dataset. For object detection experiments, please refer to the work concerning ATT-You Only Look Once (YOLO) [44]. Additionally, given the high inference speed requirements in consumer electronics surface defect detection, we selected four classic convolution-based segmentation models—DeepLabV3+, YOLOv8-Seg, U-Net, and a fully convolutional network (FCN)—to balance segmentation accuracy and computational efficiency. These models are suitable for real-world industrial applications.

#### 2.4.1. Rationale for Using Convolution-Based Segmentation Models

Transformer-based networks perform well when trained on large datasets. However, in consumer electronics surface defect detection, acquiring sufficient training data is often challenging. Additionally, real-time inference speed is a critical requirement in practical surface defect detection applications involving consumer electronics.

In manufacturing scenarios, each production line operates under strict production quotas. The time interval between the production of two consecutive products is referred to as the cycle time. The inference speed of the deployed model must remain within this cycle time, while also accounting for additional processes such as sampling and image capture. As a result, even models with high accuracy may be unsuitable for real-world deployment if they cannot meet cycle-time constraints.

In this study, we conducted a series of experiments to evaluate whether several existing segmentation methods—originally developed using general-purpose datasets, designed for few-shot learning, or widely adopted in industrial applications—could be effectively transferred to consumer electronics surface defect detection. Specifically, we examined whether these methods could deliver satisfactory predictive performance without requiring architectural modifications tailored to this domain. To this end, four representative convolution-based segmentation models—DeepLabV3+, YOLOv8-Seg, U-Net, and FCN—were selected as performance benchmarks. These models serve as comparative references for evaluating the generalizability and effectiveness of widely used segmentation approaches in real-world defect detection tasks, offering valuable insights for both academic research and practical deployment.

#### 2.4.2. The Selected Segmentation Models

##### DeepLabV3+

DeepLabV3+ [42] was proposed as an enhanced deep learning model for semantic image segmentation that integrates spatial pyramid pooling with an encoder–decoder structure. Semantic segmentation aims to classify each pixel in an image. Traditional methods typically employ either spatial pyramid pooling to capture multiscale information or encoder–decoder structures to refine object boundaries. DeepLabV3+ combines both approaches to improve segmentation accuracy. Specifically, DeepLabV3 was extended by incorporating an encoder–decoder structure. The encoder module (DeepLabV3) extracts rich semantic features using atrous convolution to preserve spatial resolution while expanding the receptive field. The decoder module refines object boundaries, addressing the loss of fine details during high-level feature extraction. The optimization strategies used in DeepLabV3+ include the following:

1. Atrous (dilated) separable convolution is applied in both the atrous spatial pyramid pooling (ASPP) and decoder modules to improve computational efficiency.
2. The Xception model [45,46,47] is adopted to enhance accuracy and speed.
3. The framework allows control over the resolution of extracted features to balance model precision and computational cost.

DeepLabV3+ has achieved state-of-the-art segmentation accuracy, with mean IoUs of 89.0% on the PASCAL VOC 2012 dataset [19] and 82.1% on the Cityscapes dataset [48].

DeepLabV3+ has been widely applied to target segmentation tasks across various domains. In [49], it was used to automatically identify and count platelets at different activation stages in electron microscopy images, achieving an error rate below 20%. In [50], DeepLabV3+ was employed to extract spatial features from hyperspectral images (HSIs), which were subsequently fused with spectral features for hyperspectral image classification. The experimental results demonstrated that the proposed framework outperformed most traditional machine learning and deep learning methods. Furthermore, in [51], the DeepLabV3 network was adapted for semantic segmentation of remote sensing images in complex environments. By replacing the backbone network with the lightweight MobileNetV2 and substituting atrous convolution with hybrid dilated convolution (HDC), the modified model maintained high segmentation accuracy while significantly reducing its parameter size and computational complexity.

##### YOLOv8-Seg

YOLOv8-Seg [43] is an advanced instance segmentation model based on the YOLOv8 architecture, designed to efficiently detect and segment objects in images. It extends the traditional YOLO object detection framework by incorporating segmentation capabilities, enabling pixel-level object recognition while maintaining real-time performance. The key features of YOLOv8-Seg are as follows:

1. Unified detection and segmentation: YOLOv8-Seg integrates object detection and segmentation into a single architecture. Unlike traditional segmentation models requiring separate processing pipelines, it performs bounding-box regression and mask prediction simultaneously, improving efficiency.
2. Efficient architecture: YOLOv8-Seg uses a modified CSP-Darknet backbone [52], optimized for lightweight, high-speed inference processes. It employs a neck structure combining feature pyramid networks (FPNs) [53] and path-aggregation networks (PANs) [54] to enhance multiscale feature representations, increasing robustness for objects of varying sizes.
3. Convolutional mask prediction: Rather than relying on transformer-based approaches, YOLOv8-Seg employs a convolutional mask prediction head, which is more computationally efficient. Segmentation masks are generated using a dynamic kernel-based approach, with each detected object assigned a segmentation kernel.
4. Loss function optimization: The model uses a combination of CIoU loss [55] for bounding-box regression and binary cross-entropy loss for mask prediction, ensuring accurate localization and high-quality segmentation.
5. Real-time performance: YOLOv8-Seg achieves real-time processing, making it suitable for applications such as autonomous driving, medical image analysis, video surveillance, and augmented reality.

YOLOv8-Seg has been widely applied in various domains. For example, in [56], the YOLO-HV model was proposed for segmenting intracerebral hemorrhages (ICHs), using YOLOv8-Seg as a base and incorporating a coordinate attention mechanism to enhance spatial feature extraction. A lightweight group normalization-based detection head (LGND) was also introduced to replace the original detection head, improving localization and classification performance. In [57], YOLO-DentSeg, a lightweight segmentation model based on YOLOv8-Seg, was developed to detect lesion areas in dental imaging. Furthermore, in [58], YOLOv8-Seg was used to detect road infrastructure defects, achieving an mAP50 of 87%, demonstrating accurate defect localization and segmentation.

YOLOv8-Seg offers a strong balance between accuracy and efficiency, making it a preferred choice for real-time segmentation tasks. Benchmarked on COCO [18] and other instance segmentation datasets, it demonstrates competitive performance compared to traditional segmentation models such as Mask R-CNN [59] while achieving significantly faster inference.

##### U-Net

U-Net [40] is a CNN architecture originally designed for semantic segmentation, particularly in medical image analysis. It was introduced by Olaf Ronneberger et al. in 2015 in their paper “U-Net: Convolutional Networks for Biomedical Image Segmentation.” U-Net has since been widely adopted in various segmentation tasks due to its high accuracy, efficient learning with limited labeled data, and ability to capture both local and global features. It has also been extensively improved in recent years, as shown in [60,61,62,63,64].

U-Net follows an FCN structure and consists of two main components: A contracting path (encoder) on the left side of the network extracts features through multiple convolutional layers followed by max pooling, thereby reducing spatial resolution while increasing feature depth. Standard rectified linear unit (ReLU) activation and batch normalization stabilize training. An expanding path (decoder) on the right side of the network reconstructs the spatial resolution using up-convolutions (transposed convolutions) and skip connections to recover fine details. The skip connections link encoder layers and decoder layers at corresponding levels, preserving spatial information and mitigating information loss.

U-Net offers several advantages:

1. High accuracy with limited data: Designed for medical imaging, where labeled data are scarce, U-Net effectively learns from small datasets using data augmentation.
2. Effective use of skip connections: Unlike standard FCNs, U-Net combines low-level and high-level features to recover fine spatial details, improving boundary segmentation.
3. Fully convolutional architecture: The absence of fully connected layers makes the model efficient and compatible with various input sizes.
4. Fast training and inference: Its relatively simple architecture enables fast convergence and real-time inference in some applications.

While U-Net has been widely used in medical image segmentation [65], its flexibility extends to industrial and manufacturing inspection, particularly for detecting defects in products and materials. Several studies have leveraged U-Net-based architectures, achieving state-of-the-art performance in specific industrial applications. For example, eSwin-UNet [66] combines the strengths of CNNs and transformers for industrial surface defect segmentation, improving both accuracy and robustness. Similarly, another study used a custom U-Net tailored for surface defect detection in industrial products, aiming to enhance manufacturing efficiency and reduce manual inspection costs.

In additive manufacturing (AM), U-Net has been applied to automatically segment pores and cracks in X-ray computed tomography (XCT) images [67]. An image-enhanced U-Net [68] has also been proposed for detecting window frame defects under varying lighting conditions and environmental settings. This framework incorporates image enhancement, data augmentation, and U-Net-based models to improve detection performance. Finally, TLU-Net [69], an innovative transfer learning-based U-Net variant, was developed to automatically detect steel surface defects. The study also analyzed the impact of different encoder choices on segmentation accuracy, providing valuable insights into model optimization for industrial applications. These studies collectively highlight U-Net’s adaptability beyond medical imaging, demonstrating its effectiveness in industrial quality control and defect detection tasks.

##### FCNs

FCNs [41] are deep learning architectures specifically designed for semantic segmentation, where each pixel in an image is assigned a class label. Introduced by Jonathan Long, Evan Shelhamer, and Trevor Darrell in their 2015 paper “Fully Convolutional Networks for Semantic Segmentation,” the original FCN transformed traditional classification-based CNNs into a fully convolutional framework, enabling end-to-end pixelwise classification without relying on fully connected layers.

An FCN replaces the fully connected layers in a CNN with convolutional layers, allowing the model to accept input images of arbitrary sizes and generate corresponding segmentation maps. The network adopts an encoder–decoder structure: the encoder extracts semantic features, and the decoder progressively restores the spatial details of the input image using upsampling techniques such as transposed convolutions. A key innovation in FCNs is the introduction of skip connections, which combine low-level spatial details from earlier layers with high-level semantic features from deeper layers. This enhances segmentation accuracy, particularly for fine object boundaries and small structures. FCNs are fully trainable end-to-end on raw images and do not require complex postprocessing operations. Unlike traditional CNNs that require fixed input dimensions, FCNs can process images of various sizes and produce dense prediction maps. By eliminating fully connected layers, FCNs reduce computational costs and offer faster inference than earlier segmentation models.

FCNs have also been widely applied across various domains. For example, in [70], a gesture segmentation method for complex backgrounds was proposed using an FCN combined with a CBAM-ResNet50 framework. Specifically, the FCN’s backbone was restructured using a modified ResNet50 model, and a convolutional block attention module (CBAM) was integrated into the residual blocks to enhance the model’s ability to extract multiscale contextual features. In [51], an FCN was applied to building contour extraction. The study introduced ME-FCN, a model designed to perceive and optimize multiscale features, effectively addressing the challenge of extracting building contours from complex remote sensing images.

The four segmentation models described above—FCN, DeepLabV3+, U-Net, and YOLOv8-Seg—are widely used for semantic and instance segmentation tasks, each model offering specific strengths and weaknesses. A detailed comparison of these models is presented in Table 6.

We compared the accuracy, speed, and computational complexity levels of these models. DeepLabV3+ achieved the highest performance in accuracy due to its multiscale feature extraction and atrous convolution. U-Net also performed well, particularly in medical and industrial applications. YOLOv8-Seg was the fastest model, making it suitable for real-time applications. FCN was relatively fast but lacked the ability to segment fine details.

DeepLabV3+ was the most computationally complex model due to its ASPP module, whereas FCN and YOLOv8-Seg were more lightweight.

The strengths and weaknesses of these models are summarized in Table 7. Each model serves different use cases, and selecting the appropriate model depends on the required accuracy, speed, and computational constraints.

#### 2.4.3. Experimental Design

In our experiments, the LCFC-Laptop dataset was divided into training, validation, and test sets in a 7:2:1 ratio. The training set contained 10,382 images, the validation set 2966 images, and the test set 1484 images. We conducted fair evaluations on four selected segmentation models. The corresponding numbers of parameters and floating-point operations (FLOPs) for each model are also provided as references for the research community in assessing model performance on consumer electronics surface defect detection datasets.

As these four models are widely recognized for their neural network designs, this study provides additional insights to support the development of improved models for real-world datasets. To ensure a fair comparison, no hyperparameter tuning was applied; all models were trained using their default settings. The mIoU metric, defined in Equations (1) and (2), was used to evaluate the models. In Equation (1), TP, FP, and FN denote true positives, false positives, and false negatives.

(1) $${\mathrm{IoU}}_{i}=\frac{\mathrm{TP}}{\mathrm{TP}+\mathrm{FP}+\mathrm{FN}}$$

(2) $$\mathrm{mIoU}=\frac{\sum_{i=1}^{N}{\mathrm{IoU}}_{i}}{N}$$

Four deep learning-based segmentation networks were evaluated:

1. DeepLabV3+: Used ResNet50 as the backbone with input image dimensions of 1024 × 1024. During training, data augmentation techniques, including random resizing, cropping, and flipping, were applied using preset ratios. The model was trained using stochastic gradient descent (SGD) with a learning rate of 0.01, momentum of 0.9, and batch size of 2.
2. FCN: Also used ResNet50 as the backbone, with input image dimensions of 1024 × 1024. The same data augmentation techniques—random resizing, cropping, and flipping—were applied. Training was performed using SGD with a learning rate of 0.01, momentum of 0.9, and batch size of 2.
3. U-Net: Used the U-Net-S5-D16 architecture as the backbone, with an FCN head as an auxiliary head, and an input image size of 1024 × 1024. Data augmentation techniques included random resizing, cropping, and flipping. The model was trained using SGD with a learning rate of 0.01, momentum of 0.9, and batch size of 2.
4. YOLOv8-Seg: Used the YOLOv8-X model as the backbone, with an input image size of 1280 × 1280. Data augmentation strategies included Mosaic augmentation, random scaling, and cropping. Training was performed using SGD with a learning rate of 0.01, momentum of 0.937, and batch size of 2.

The hyperparameters of the four models are shown in Table 8.

## 3. Results

**Figure 7:**
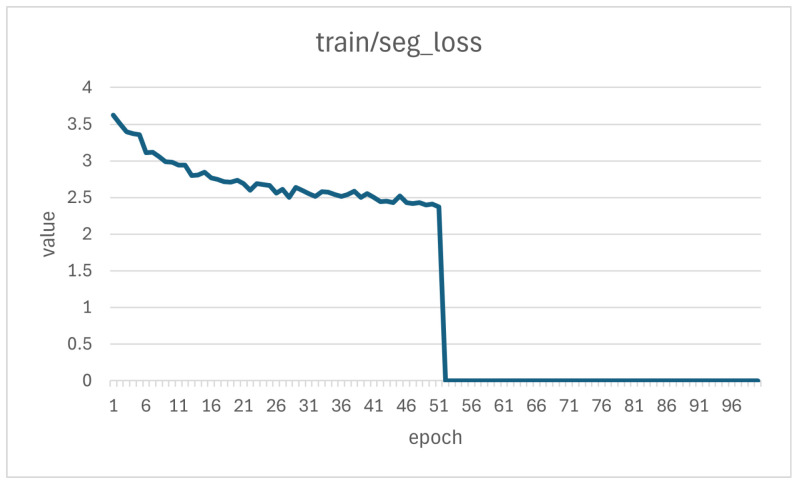
Loss variation during training.

**Figure 8:**
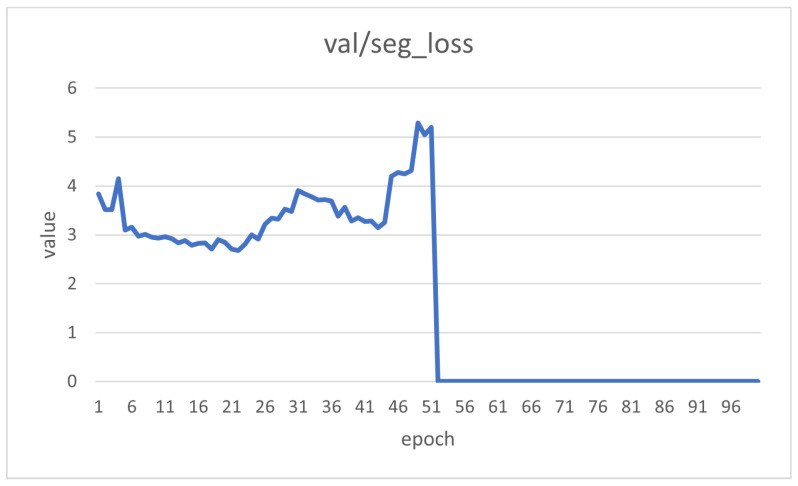
Loss variation during validation.

We conducted training, validation, and testing, as shown in Table 9, Table 10 and Table 11. YOLOv8-Seg achieved mIoU scores of 0.73, 0.64, and 0.60 on the training, validation, and test sets, respectively. To account for the inherent randomness of each method, we conducted each experiment three times. The average standard deviation across these runs was 0.001. Although minor variations occurred, the overall results remained stable. DeepLabV3+ achieved mIoU scores of 0.51, 0.52, and 0.52 on the same sets. FCN achieved mIoU scores of 0.60, 0.61, and 0.59, respectively, whereas U-Net achieved mIoU scores of 0.41, 0.46, and 0.41, respectively. Overall, YOLOv8-Seg, with a relatively lightweight model size (11.78 M), delivered the best performance on the independent test set. Nevertheless, none of the four models achieved the accuracy required for practical deployment on production lines. This study also analyzed the learning curves, comparing the model’s training and validation loss trends, as shown in Figure 7 and Figure 8.

Hyperparameter tuning is an important aspect of model training. In our earlier experiments (Table 9, Table 10 and Table 11), all models were trained, validated, and tested using their default settings to ensure a fair comparison. However, to demonstrate the potential impact of hyperparameter optimization, we conducted a grid search for YOLOv8-Seg on the dirt defect category (Table 12). The results showed a significant 15.4% improvement in mIoU compared to the baseline. We therefore recommend that users consider moderate hyperparameter tuning, when computational resources permit, to further improve model performance.

**Figure 9:**
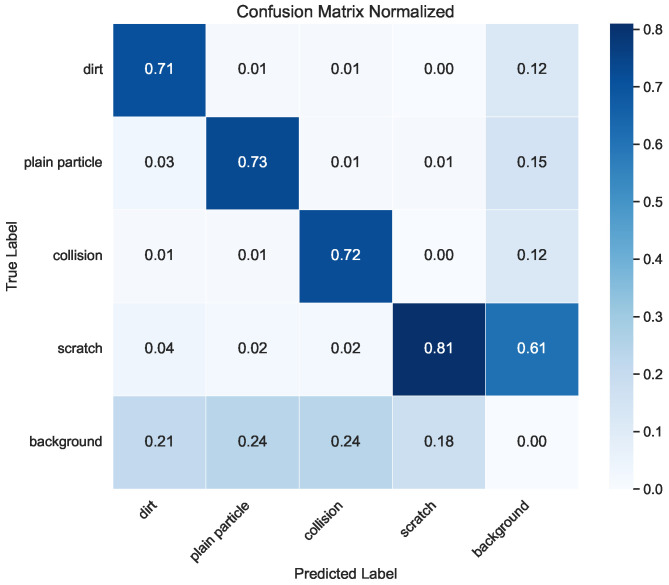
Confusion matrix of the YOLOv8-Seg model. The x-axis denotes the ground-truth classes, and the y-axis denotes the predicted classes. Each row corresponds to a true class, and each column to a predicted class.

In addition to the mIoU results, this study presents the precision, recall, and confusion matrix of the YOLOv8-Seg model to provide a more comprehensive understanding of its performance (Table 13 and Figure 9). To analyze the effect of U-Net’s skip connections on samples with varying levels of clarity, the LCFC-Laptop dataset was divided into three categories: clear, mildly blurred, and heavily blurred. We then compared U-Net’s performance with that of the more recent YOLOv8-Seg model across these clarity levels. The results are shown in Table 14.

As shown in Table 15, this study compared the impact of unknown samples on model performance through a binary segmentation experiment. Two setups were designed: one model was trained with unknown defect samples included, and the other excluded them. The results demonstrate that the model trained without unknown samples outperformed its counterpart. However, in real-world data collection scenarios, the presence of unknown defects is unpredictable and cannot be controlled in advance.

As the dataset used in this study includes a considerable number of small samples, we further investigated different feature fusion strategies by comparing three feature pyramid architectures: PAN, FPN, and PAN+FPN. The experimental results, shown in Table 16, demonstrate that the combined FPN+PAN feature fusion strategy yielded the best performance on the LCFC-Laptop dataset.

In this study, the surface defects to be detected often occupy only a very small portion of the input image. For instance, in a 5000 × 5000-pixel image, the defect region may be as small as 25 pixels in area. To address this, we investigated whether incorporating an attention mechanism for feature enhancement could improve the detection of such subtle regions. As shown in Table 17, the experimental results demonstrate that integrating an attention mechanism into the backbone network significantly enhanced relevant features, thereby improving detection performance.

**Figure 10:**
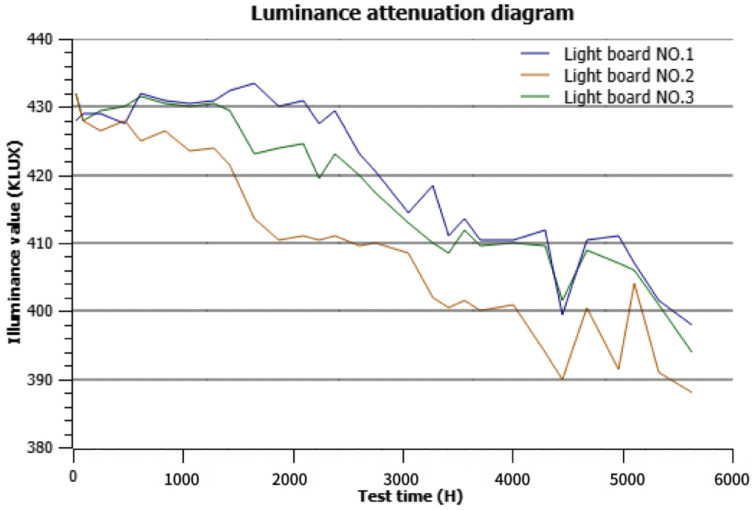
Illumination system light decay experiment. The x-axis represents time (hours), and the y-axis represents brightness. After 6000 h of testing, light intensity degradation did not exceed 40%.

To evaluate the robustness of the illumination system, we conducted a series of controlled experiments. Since the illumination system is enclosed in a sealed black box, it is unaffected by ambient indoor lighting. However, light decay over time is inevitable (Figure 10). To simulate this degradation, we tested the dirt category using the YOLOv8-Seg model under three lighting conditions: baseline (original brightness), −20% brightness, and −40% brightness. As shown in Table 18, the highest mIoU was 78.7%, and the lowest was 78.1%, with a variation of less than 1%. These results demonstrate that the proposed illumination system is reasonably robust against significant brightness fluctuations.

**Figure 11:**
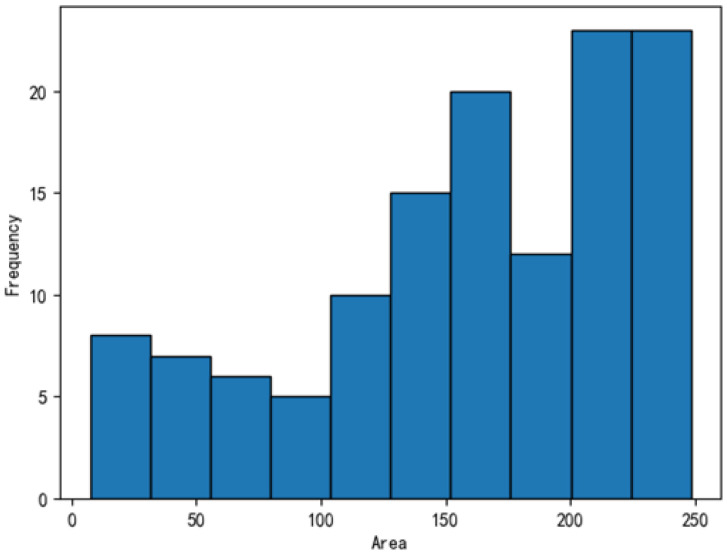
Frequency distribution of dirt defect areas.

**Figure 12:**
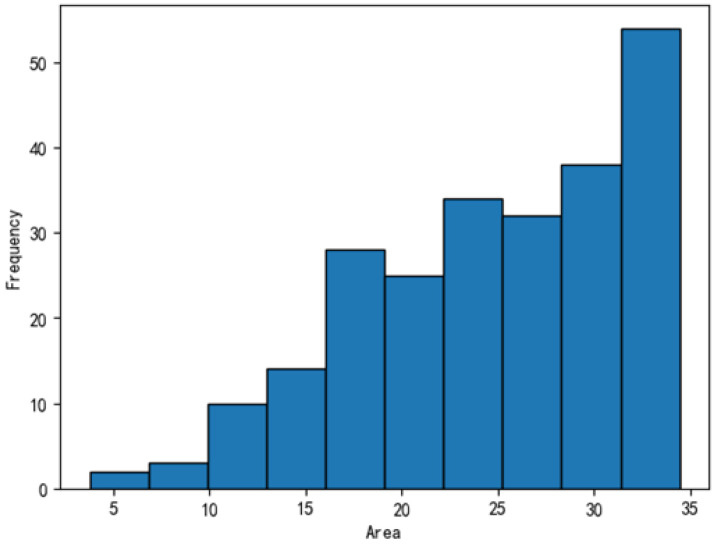
Frequency distribution of plain-particle defect areas.

**Figure 13:**
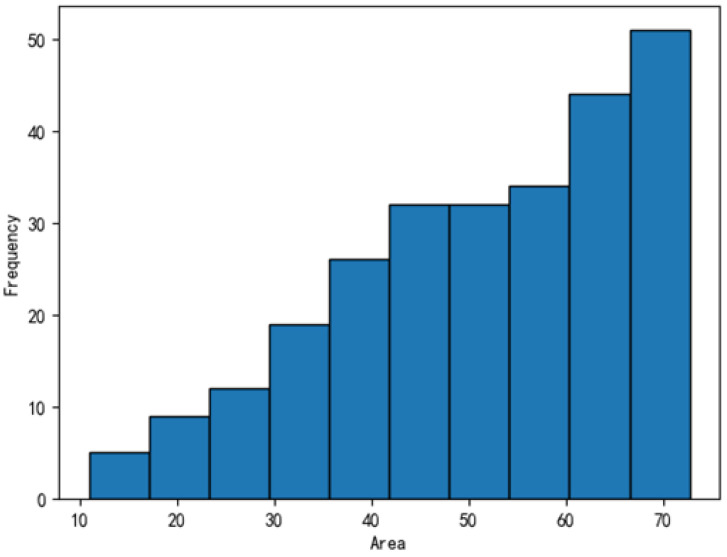
Frequency distribution of collision defect areas.

**Figure 14:**
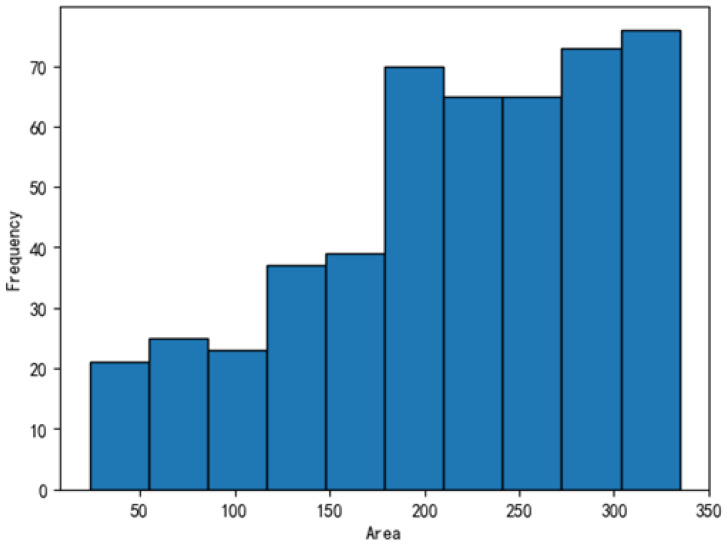
Frequency distribution of scratch defect areas.

The dataset was further analyzed, with the defect distributions illustrated in Figure 11, Figure 12, Figure 13 and Figure 14. In these figures, the areas of the top 95% most frequently occurring defects were grouped into 10 bins to generate frequency distribution histograms. The x-axis represents area intervals, and the y-axis indicates frequency. For the dirt and scratch categories, 95% of defects had widths and heights under 60 pixels, classifying them as small objects relative to a 1000-pixel image. Moreover, over 97% of these defects were smaller than 40–50 pixels in both width and height, significantly affecting segmentation performance. The other two defect types were even smaller, typically less than 8 pixels in both dimensions. Further analysis of the annotated images revealed that plain-particle defects tended to be elongated and narrow, with widths often smaller than 8 pixels. As a result, these defects were more likely to be lost during the downsampling process in each network, further reducing segmentation accuracy.

**Figure 15:**
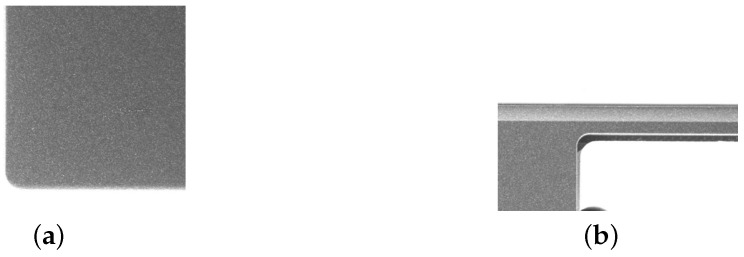
Examples of ambiguous defects. (**a**) Ambiguous defect example 1. (**b**) Ambiguous defect example 2.

**Figure 16:**
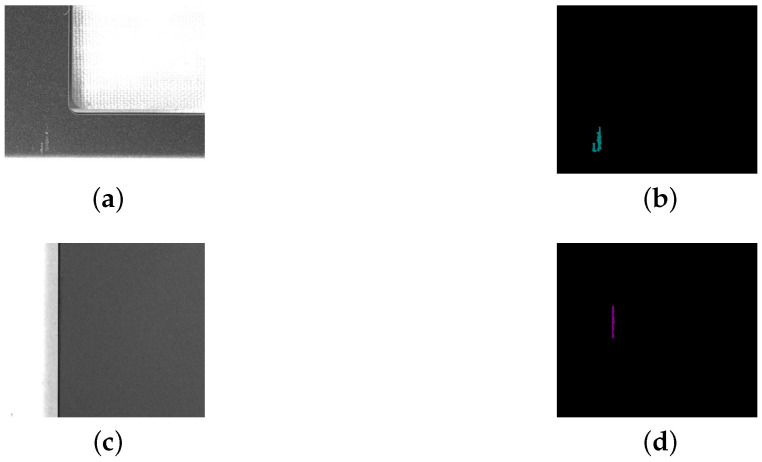
Examples of visual similarities between different categories. (**a**) Original image—dirt. (**b**) Labeled image—dirt. (**c**) Original image—scratches. (**d**) Labeled image— scratches.

**Figure 17:**
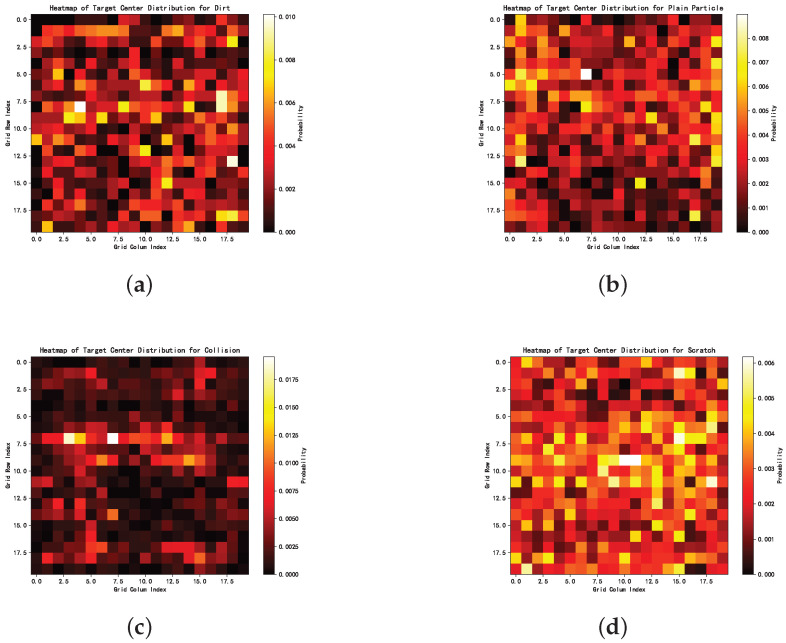
Heatmaps of defect location distributions for the four categories. (**a**) Dirt. (**b**) Plain particles. (**c**) Collisions. (**d**) Scratches.

In addition, consumer electronics surface defect detection posed several major challenges:

1. Blurry defects: As shown in Figure 15, the contrast between defects and their backgrounds was relatively low, making them difficult to distinguish with the naked eye and often resulting in missed annotations. Segmentation models also tended to perform poorly on such low-contrast defects.
2. Visual similarity between defect categories: As illustrated in Figure 16, different types of defects were visually similar, leading to frequent misclassifications. For example, thin, elongated dirt marks can closely resemble scratches, making the two difficult to distinguish.
3. Randomized spatial distribution: As shown in the heatmaps in Figure 17, defect locations were distributed randomly across the images, without any clear pattern. This randomness may have further increased the convergence difficulty of the networks.

In these heatmaps, the x-axis represents column coordinates, the y-axis indicates row coordinates, and the color bar on the right displays the probability of defect occurrence. To generate these heatmaps, we calculated the center point of each defect and divided each image into a 20 × 20 grid. Each defect’s center point was then mapped to the corresponding grid cell. By counting the number of defects of each category within each cell and visualizing the associated frequency, we produced heatmaps representing the probability distribution of defect categories across image locations. Figure 15, Figure 16 and Figure 17 provide qualitative insights into the dataset. To support these findings with quantitative information, we conducted statistical analyses of defect length, area, and perimeter across different categories, with the results summarized in Table 19, Table 20 and Table 21.

To further analyze the occurrence of false positives and false negatives in real-world production scenarios, this study identified the most commonly misclassified categories based on actual production line data and provided possible explanations for their causes.

False Negatives: Particles Tend to be Missed

- Particle Characteristics—Defective particles are typically small, irregularly shaped objects such as dust, metal shavings, plastic debris, or coating residues. Although generally small, their shape and color may sometimes contrast with the C-cover surface, affecting visual appearance.
- Particle Location—Particles usually appear on the exposed areas of the C-cover, such as the top panel, bottom, and edges, and tend to accumulate in frequently contacted areas like corners and seams.
- Significance:
  − Aesthetic Degradation: The presence of particles reduces product value and compromises appearance, reducing its market appeal.
  − Visual Quality Impact: Particles negatively affect perceived quality. Visual imperfections may result in customer rejection, especially when appearance is a key purchasing factor.
  − Coating and Surface Treatment: During processes such as spraying and coating, particles can cause uneven coverage, reduced adhesion, bubbling, or peeling, thereby diminishing durability and scratch resistance.

False Positives: Acceptable Particles (e.g., 0.1 mm) and Airborne Lint or Dust

- Significance—In production environments, the detection of particles within the acceptable tolerance range suggests that the current quality control criteria already allow for a certain degree of leniency. However, continued monitoring is essential to prevent future quality degradation. Ensuring that the frequency of such particles does not increase over time is critical to avoiding a gradual rise in defect rates that may eventually exceed acceptable limits.

## 4. Future Work

One direction for future work involves expanding the current dataset—which currently focuses on laptops—to include defect data from a broader range of consumer electronics, such as smartphones and tablets. While laptops are emphasized due to their prominence in Lenovo’s production portfolio, the extended dataset will incorporate devices made from diverse materials commonly found in modern electronics, including plastic, metal, and graphene. All new data will be annotated following the same rigorous standards used on the production line to ensure consistency and industrial relevance. Another objective involves addressing the lower performance observed in certain categories within the current dataset when applying existing object detection and segmentation methods. This includes developing new approaches capable of better detecting challenging samples, which are often characterized by ambiguity, undefined defect types, and varied size distributions—traits that differ significantly from those in general-purpose object detection and segmentation datasets.

A further critical area of exploration is balancing inference speed with accuracy, ensuring that models can be deployed effectively on production lines with strict cycle-time requirements for real-time surface defect detection. Current segmentation algorithms developed using general-purpose datasets still require significant improvements to meet the demands of real-world surface defect detection. As a result, general segmentation methods cannot be directly applied to consumer electronics surface defect detection tasks.

This study contributes by providing a publicly accessible, real-world industrial dataset, offering an important reference for both academic and industrial communities to develop segmentation and object detection algorithms that are applicable to actual manufacturing environments.

## 5. Conclusions

This study presents the first dataset comprising real-world surface defect samples specifically from consumer electronics, with annotations rigorously curated and repeatedly validated according to actual production line standards. The dataset includes both bounding boxes for object detection and pixel-level masks for semantic segmentation tasks.

A comprehensive evaluation using several widely adopted semantic segmentation models revealed that current methods still exhibit notable performance limitations when applied to this dataset. These findings underscore the challenges of real-world defect detection and provide a valuable benchmark for both industrial practitioners and academic researchers seeking to develop more effective, application-oriented solutions.

Looking ahead, we aim to expand the dataset to include a broader range of materials and device types. In parallel, we will focus on designing more efficient models that meet the strict real-time constraints of industrial deployment. We hope this work serves as a foundation for future advancements in surface defect detection within the consumer electronics industry.

## Figures and Tables

**Table 1:** Comparison between the LCFC-Laptop and MVTec AD datasets.

	LCFC-Laptop	MVTec AD
Number of defects	14,478	5354
Number of lighting sources	6	1
Application	Surface defect detection in consumer electronics	Industrial defect detection
Annotation	Pixel-precise ground truth, object detection	Pixel-precise ground truth
Evaluation	Yes	Yes
Original resolution	5000×5000	700×700–1024×1024

**Table 2:** Statistics for the object detection task.

Defect Type	Number of Defects
Dirt	11,285
Plain particles	605
Edge particles	35
Collisions	29
Scratches	1104
Unknown	1420
All	14,478

**Table 3:** Statistics for the segmentation task.

Defect Type	Number of Defects
Dirt	2572
Plain particles	4788
Collisions	5265
Scratches	9868
All	22,512

**Table 4:** The six lighting combinations comprise 35 specific white and blue light sources with varying incident angles and model numbers, each corresponding to a designated position in the PCL system.

Color	Model	Angle (°)
White	M132	0
Blue	M133	0
White	M134	0
White	M123	30
Blue	M124	30
White	M125	45
Blue	M126	45
White	M127	60
Blue	M128	60
White	M129	90
Blue	M130	90
Blue	M131	90
White	M100	30
Blue	M101	30
White	M102	45
Blue	M103	45
White	M105	90
Blue	M106	90
White	M106	90
Blue	M107	90
White	M108	30
Blue	M109	30
White	M110	45
Blue	M111	45
White	M112	90
Blue	M113	90
Blue	M114	90
White	M115	30
Blue	M116	30
White	M117	45
Blue	M118	45
White	M119	45
White	M120	90
Blue	M121	90
Blue	M122	90

**Table 5:** Wavelengths and brightness levels of lighting sources.

	Wavelength (nm)	Brightness (Luminous Flux)
White	Red 620–630 nm U +Blue 465–475 nm U +Green 520–530 nm	0–130,000
Red	620–630 nm	0–130,000
Blue	465–475 nm	0–130,000

**Table 6:** Performance comparison of FCN, DeepLabV3+, U-Net, and YOLOv8-Seg.

Model	Accuracy (mAP@50 on COCO)	Inference Speed (FPS on RTX 3090)	Computational Complexity
FCN	$\tilde{6}5\%$	30+ FPS	Low
DeepLabV3+	$\tilde{8}2\%$	10–20 FPS	High
U-Net	$\tilde{8}0\%$	15–25 FPS	Medium
YOLOv8-Seg	$\tilde{7}8\%$	50+ FPS	Low–Medium

**Table 7:** The strengths and weaknesses of the four selected models.

Model	Strengths	Weaknesses
FCN	- Simple and efficient - Works with variable input sizes - End-to-end trainable	- Lower segmentation accuracy - Lacks fine-grained details
DeepLabV3+	- High segmentation accuracy - Strong boundary preservation - Performs well on complex datasets	- Slow inference speed - High computational cost
U-Net	- Suitable for medical and industrial segmentation - Strong boundary refinement - Performs well on small datasets	- High memory consumption - Not optimized for real-time use
YOLOv8-Seg	- Real-time performance - Combines object detection and segmentation - Efficient on edge devices	- Lower accuracy than DeepLabV3+ and U-Net - May struggle with fine-grained segmentation

**Table 8:** Hyperparameters of the four selected models.

Model	Batch Size	Epochs	Learning Rate	Optimizer
YOLOv8-Seg	2	100	0.01	SGD
Mask R-CNN	2	100	0.01	SGD
DeepLabV3+	2	100	0.01	SGD
U-Net	2	100	0.01	SGD

**Table 9:** Training performance of the four selected segmentation models.

	YOLOv8-Seg	DeepLabV3+	FCN	U-Net
Number of parameters	11.78 M	41.22 M	47.13 M	28.99 M
FLOPs	0.04 T	0.71 T	0.79 T	0.81 T
IoU (dirt)	0.74	0.50	0.59	0.42
IoU (plain particles)	0.83	0.49	0.61	0.41
IoU (collisions)	0.58	0.52	0.60	0.41
IoU (scratches)	0.77	0.52	0.59	0.41
mIoU	0.73	0.51	0.60	0.41

**Table 10:** Validation performance of the four selected segmentation models.

	YOLOv8-Seg	DeepLabV3+	FCN	U-Net
Number of parameters	11.78 M	41.22 M	47.13 M	28.99 M
FLOPs	0.04 T	0.71 T	0.79 T	0.81 T
IoU (dirt)	0.62	0.56	0.61	0.46
IoU (plain particles)	0.66	0.52	0.61	0.47
IoU (collisions)	0.60	0.49	0.60	0.47
IoU (scratches)	0.67	0.53	0.63	0.46
mIoU	0.64	0.52	0.61	0.46

**Table 11:** Test performance of the four selected segmentation models.

	YOLOv8-Seg	DeepLabV3+	FCN	U-Net
Number of parameters	11.78 M	41.22 M	47.13 M	28.99 M
FLOPs	0.04 T	0.71 T	0.79 T	0.81 T
IoU (dirt)	0.60	0.52	0.59	0.41
IoU (plain particles)	0.62	0.49	0.58	0.40
IoU (collisions)	0.49	0.53	0.60	0.40
IoU (scratches)	0.68	0.52	0.59	0.41
mIoU	0.60	0.52	0.59	0.41

**Table 12:** Results of hyperparameter grid search for YOLOv8-Seg on the dirt defect category.

Epoch	Learning Rate	Optimizer	mIoU
150	0.01	SGD	0.597
150	0.02	SGD	0.751
100	0.01	SGD	0.704
150	0.01	Adam	failed to converge
100	0.02	SGD	0.763
100	0.01	Adam	failed to converge
150	0.02	Adam	failed to converge
100	0.02	Adam	failed to converge

**Table 13:** Precision and recall of the YOLOv8-Seg model.

	Precision	Recall
Dirt	0.716	0.677
Plain particles	0.717	0.614
Collisions	0.706	0.599
Scratches	0.769	0.769

**Table 14:** Performance of U-Net and YOLOv8-Seg under varying visual conditions.

	U-Net	YOLOv8-Seg
Clear	0.413	0.751
Mildly blurred	0.399	0.732
Heavily blurred	0.379	0.691

**Table 15:** Comparison of binary segmentation results with and without unknown samples.

	mIoU
Without unknown samples	73.4%
With unknown samples	70.6%

**Table 16:** Performance comparison of various feature pyramid architectures for feature fusion.

	mIoU
FPN	73.2%
PAN	65.6%
PAN+FPN	78.4%

**Table 17:** Comparison of the performance of YOLOv8-Seg with and without an attention mechanism on the LCFC-Laptop dataset.

	mIoU
Backbone with attention	71.2%
Backbone without attention	70.6%

**Table 18:** Robustness test of the illumination system.

	mIoU
Baseline	78.4%
−20% brightness	78.7%
−40% brightness	78.1%

**Table 19:** Statistical summary of defect areas in the LCFC-Laptop dataset.

	Plain Particles	Collisions	Dirt	Scratches
Mean area (pixels^2^)	251.65	679.86	5101.20	1867.24
Standard deviation of area (pixels^2^)	411.05	61,205.08	67,441.41	2848.38

**Table 20:** Statistical summary of defect perimeters in the LCFC-Laptop dataset.

	Plain Particles	Collisions	Dirt	Scratches
Mean perimeter (pixels)	58.40	118.45	332.87	299.52
Standard deviation of perimeter (pixels)	42.42	98.13	241.24	177.77

**Table 21:** Statistical summary of defect lengths in the LCFC-Laptop dataset.

	Plain Particles	Collisions	Dirt	Scratches
Mean defect length (pixels)	16.09	41.15	147.07	118.18
Standard deviation of defect length (pixels)	25.20	49.71	131.23	82.91

## Data Availability

As the dataset used in this study includes unreleased products and is subject to copyright restrictions, it is not publicly available. However, it can be provided to interested readers upon reasonable request.
